# Pleural empyema due to *Salmonella* in a patient with bronchogenic carcinoma: the first case report from a cancer hospital in Egypt

**DOI:** 10.1099/acmi.0.000151

**Published:** 2020-07-15

**Authors:** Ahmed Samir Abdelhafiz, Mona Wassef, Mohamed Alorabi

**Affiliations:** ^1^​ Department of Clinical pathology, National Cancer Institute, Cairo University, Cairo, Egypt; ^2^​ Department of Clinical pathology, Shefaa Al Orman Hospital, Luxor, Egypt; ^3^​ Department of Clinical and Chemical Pathology, Faculty of Medicine, Cairo University, Cairo, Egypt; ^4^​ Department of Clinical Oncology, Faculty of Medicine, Ain Shams University, Cairo, Egypt; ^5^​ Department of Medical Oncology, Shefaa Al Orman Hospital, Luxor, Egypt

**Keywords:** *Salmonella*, empyema, cancer, bronchogenic carcinoma

## Abstract

**Background:**

*
Salmonella* species are motile, Gram-negative facultative anaerobic bacilli, which belong to the family *Enterobacteriaceae *. The most common clinical presentations of *Salmonella* infection are gastroenteritis and enteric fever. Detection of *Salmonella* organisms in empyema is very rare.

**Case presentation:**

We report the case of a 66-year-old female patient with bronchogenic carcinoma who developed empyema, and *Salmonella* was identified from the culture of pleural fluid. After antimicrobial therapy and other therapeutic measures, including the insertion of an intercostal tube, oxygen supplementation, frequent suction of respiratory secretions, and chest physiotherapy, the patient's condition improved. To the best of our knowledge, this is the first case to be reported in Egypt.

**Conclusions:**

Our case sheds light on the role of *Salmonella* in immunocompromised patients in general and cancer patients in specific. We recommend further study of this role, since it may lead to a better understanding of the pathogenicity of this organism in these patients.

## Introduction

*Salmonella* species were first described in the 1880s by Salmon and Smith, and it was named after Daniel E. Salmon who isolated *Salmonella cholerasuis* from pigs [1, 2]. *Salmonella* are motile, non-spore-forming Gram-negative facultative anaerobic bacilli, which belong to the family *Enterobacteriaceae* [2]. The most common clinical presentations of infection are gastroenteritis and enteric fever. However, *Salmonella* can also cause bacteraemia and manifest in the form of enteric or urinary carrier states [1, 3]. While chronic carrier state may develop in less than 1 % of patients with non-typhoidal *Salmonella* infection, bacteremia may develop in up to 8% of patients, especially the vulnerable groups, including extremes of age, and immunocompromized patients [4]. Outside the gastrointestinal tract (GIT), *Salmonella* infection is quite uncommon, and the development of focal pulmonary infection, including empyema, occurs in less than 1 % of patients [4]. It is hypothesized that empyema occur in these patients through seeding from bacteraemia, or nearby sources of infection such as the pancreas or the spleen [1].

Predisposing factors for development of *Salmonella empyema* include old age and the presence of diabetes mellitus, malignancy, iron overload, chronic renal insufficiency and the presence of another pulmonary disease [3, 5]. Here we outline a case report of a female patient with bronchogenic carcinoma who developed empyema and *Salmonella* was identified as the causing organism. The case was identified at Shefaa Al Orman Hospital, a new cancer hospital established in Upper Egypt. To the best of our knowledge, this was the first case of *Salmonella empyema* to be reported in Egypt.

## Case presentation

A 66-year-old Egyptian housewife was referred to the medical oncology clinic at Shefaa Al-Orman Hospital from a general practitioner who suspected lung cancer for further assessment. Shefaa Al-Orman Hospital is the only specialized cancer center providing free cancer treatment in Luxor governorate. The referral letter of the patient stated that she had a history of a gradually worsening productive cough of white sputum and shortness of breath for a 1 month period as well as left-sided pleural effusion. She denied having fever, chills, haemoptysis, weight changes, contact with sick people or travel within that time.

On physical examination, the patient was tachypneic with peripheral oxygen saturation at 95 % at room air, bilateral rhonchi and diminished breath sounds more prominent on the left side of the chest by lung auscultation, and there was no evidence of lymphadenopathy. The initial chest radiograph revealed left-sided mild pleural effusion. Further characterization with a chest and abdomen CT scan with intravenous contrast revealed left upper lung lobe mass about 50×52×60 mm impressive of bronchogenic carcinoma, with mild pleural effusion on the left side. CT-guided biopsy and fine-needle aspiration from the lung mass was consistent with grade 2 bronchogenic adenocarcinoma. Bone scan showed osseous lesions at the right sternoclavicular joint (history of trauma) and D11 vertebra (likely benign). However, magnetic resonance imaging (MRI) on the lumbosacral region showed multiple sclerotic lesions, while brain MRI was free.

Laboratory investigations, including complete blood count, kidney and liver function tests were all within normal limits. Her tumour harboured exon 19 EGFR gene mutation, which was detected using Ventana line strip assay (PCR and hybridization). The patient started to receive daily treatment with oral gefitinib (250 mg/day) as well as denosumab (120 mg, subcutaneously, every 28 days), and oral supplementation of calcium (500 mg/day), and vitamin D (one mcg/day). After 2 months from treatment, the patient reported marked decrease in the severity of respiratory symptoms and improvement in her performance status to be grade I instead of II before the treatment according to the Eastern Cooperative Oncology Group (ECOG) scale [6]. Radiological evaluation after 2 months from starting treatment revealed stable disease according to Response Evaluation Criteria in Solid Tumors (RECIST) guidelines (version 1.1) [7]. So, the decision was to continue the same treatment protocol.

**Fig. 1.**
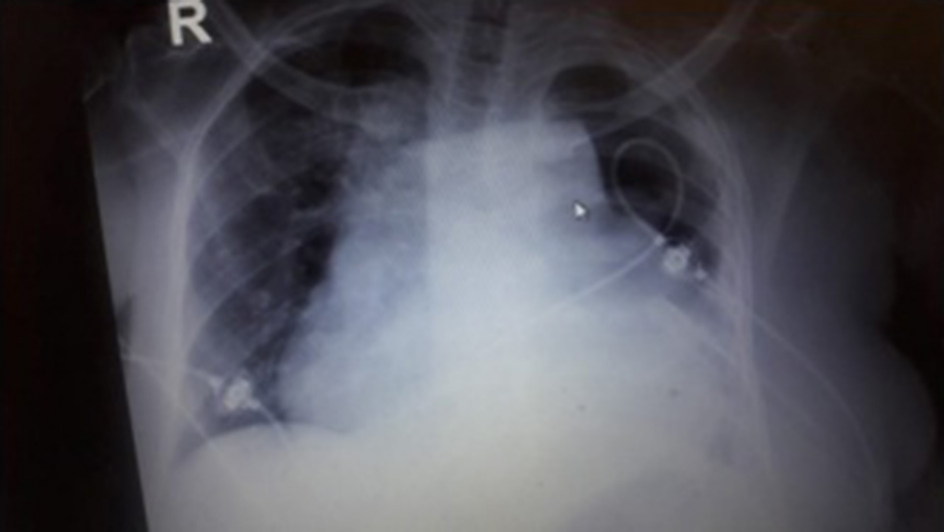
Chest x-ray of the patient showing left-sided hydro-pneumothorax.

Four months later, the patient presented to the emergency room with shortness of breath and dyspnea. A pigtail catheter was inserted for pleural effusion drainage. Unfortunately, the pigtail catheter became obstructed and slipped after 18 days and chest x-ray showed left-sided hydro-pneumothorax. Fig. 1 shows the x-ray of the patient. The medical team decided to place a chest tube thoracostomy with drainage of 500 CC of pus.

The pus sample was sent for culture and sensitivity testing. The sample was turbid and greenish in colour. It was cultured on blood agar, chocolate agar and MacConkey agar. Growth of Gram-negative bacilli was identified. Biochemical reactions, including Triple Sugar Iron (TSI) slant, which revealed alkali, TSI butt, which revealed acid, Lysine Iron Agar (LIA) (positive), Sulfur Indole Motility (SIM) (Sulfide positive, Indole negative, Motility positive), citrate (positive) and urease (negative) were done, which revealed the growth of *Salmonella* species according to the WHO laboratory protocol for biochemical identification of *Salmonella* and *Shigella* [8]. The results were confirmed using VITEK 2 COMPACT, an automated microbial identification system commonly used for microbial identification in clinical laboratories**,** which eliminates human subjectivity [9]. Using pure isolates of organisms to be tested, VITEK 2 Compact allows the identification of micro-organisms in 4 hours. The system uses fluorogenic and turbidimetric methods for organism identification and susceptibility testing, respectively [9].

In order to verify the results, samples were sent to an accredited lab at Kasr Alainy hospitals, Cairo University, which confirmed the result using the same system (VITEK 2 COMPACT). After 7 days of antimicrobial treatment including levofloxacin 750 mg IV, ceftazidime 2 gm IV, and clindamycin 600 mg IV, her condition stabilized, and she was discharged after the removal of intercostals tube, and she was still receiving her oncological treatment at the time of submission.

## Discussion

The most common clinical expression of *Salmonella* infection is acute gastroenteritis [1]. However, *Salmonella* infection can present with extra-intestinal manifestations including fever and bacteremia without enteric fever in some cases, as well as focal infections including septic arthritis, osteomyelitis, vascular infection, endocarditis, urinary tract infection and splenic abscess [3]. Clinically, bacteremia caused by *Salmonella* is characterized by the presence of a hectic fever that might last for days or weeks. *Salmonella* may also present with a chronic carrier in less than 1 % of patients [10].

Pleural empyema due to *Salmonella* infection is a rare localized infection that may follow *Salmonella bacteraemia*. Fewer than 40 cases of *Salmonella empyema* have been reported in different regions in the world [1, 10].

*Salmonella empyema* usually affects old patients or patients with conditions such as diabetes mellitus, sickle cell anemia, malignancies stemming from conditions such as lung cancer, leukemia and lymphoma, as well as patients receiving corticosteroid therapy [3, 5]. Among these risk factors, our patient was old, hypertensive and had bronchogenic carcinoma. Since *Salmonella* is an intracellular pathogen, *Salmonella* syndrome is common in patients with cellular immunodeficiency, including AIDS [3]. Treatment of those patients is usually difficult, and repeated infection is common [3].

Salmonellosis bacteremia occurs between 15 and 100 times more frequently in AIDS patients than in the general population [11, 12]. This higher frequency can be attributed to defective cell-mediated immunity, impaired B-cell function, prior use of antibiotics, and diminished gastric acidity [12–15]. Fluoroquinolone is the treatment of choice for at least 4–6 weeks [16]. However, there was a significant increase in fluoroquinolone-resistant strains obtained from HIV-infected patients [17]. Resistant strains may result from immunosuppression, and prior use of antimicrobial agents [18]. Our patient was tested and she was negative for HIV.

Many different haematopoietic cell types express the epidermal growth factor receptor (EGFR), including macrophages, monocytes, plasma cells and specific T-cell subsets such as effector CD4 T cells and FoxP3-expressing regulatory CD4 T cells (Tregs) [19–22]. Thus, EGFR antagonists used to target tumours can interfere with the function of the immune system [23]. Our patient received gefitinib as a part of her oncological treatment. Gefitinib is a selective small-molecule epidermal growth factor receptor tyrosine kinase inhibitor (EGFR TKI) indicated for the treatment of adults with locally advanced or metastatic non-small cell lung cancer (NSCLC) with activating mutations of EGFR tyrosine kinase [24]. A systematic review and meta-analysis of randomized controlled trials revealed that the use of EGFR-TKIs significantly increased the risk of developing all-grade infectious events in NSCLC patients [25]. The receptor activator of nuclear factor-κB (RANK), the RANK ligand (RANKL), and osteoprotegerin, a decoy receptor for RANK, regulate osteoclastogenesis and may play a key role in bone metastasis [26]. Denosumab is a fully human monoclonal antibody that binds to and neutralizes RANKL, inhibits osteoclast function, prevents generalized bone resorption and local bone destruction, and has become a therapeutic option for preventing or delaying skeletal-related events in different malignancies including lung cancer as in our patient [27]. On the other hand, RANKL was found to be a costimulatory cytokine for T-cell activation and lymphocyte development [28, 29]. We think that the old age, the presence of malignancy and treatment with EGFR TKI and RANKL inhibitors in our patient were associated with the development of defective cell-mediated immunity against different types of antigens.

Localized infections may occur in any site in the body in up to 10 % of patients with *Salmonella* bacteremia, and the onset of clinical presentation may be delayed [6]. The development of localized *Salmonella* infection usually results from seeding from bacteremia in patients with positive blood culture. However, due to low bacterial load, blood cultures are usually negative in *Salmonella bacteremia*, and the sensitivity of the detection of the organism from the blood decreases as the illness continues [1]. About 30 % of these cases are associated with positive blood culture, and less than 40%ae associated with positive stool cultures [1]. Diagnosis of localized *Salmonella* infection usually depends on the detection of the organism in corresponding specimens, which are normally sterile. In the case of empyema, pleural fluid is the sample, and the culture of the organism is usually done on ordinary media such as blood agar [3]. Interestingly, blood culture and nasal swab were negative in our patient. Since there is no source of nearby infection with *Salmonella *, we think that the source of infection in our patient is bacteremia, which was not detected by blood culture due to low bacterial load and/or delayed collection of the sample.

Several antimicrobial agents can be used for the treatment of *Salmonella bacteremia* or localized infection. Examples include ampicillin, chloramphenicol, trimethoprim-sulfamethoxazole as well as third-generation cephalosporins [3, 30]. *Salmonella* infection of the pleuropulmonary region has a high mortality rate. In a report by Aguado *et al*., the mortality rate was 63 % in 11 patients with this infection. However, this might be related to the high number of immune-compromised patients in their cohort [3]. Fortunately, based on the antibiotic sensitivity report, our patient received Ceftazidime, Levofloxacin and Clindamycin, and her condition improved

In conclusion, our case sheds light on the role of *Salmonella* in immunocompromised patients in general and cancer patients in specific. Although the original source of infection cannot be traced in many cases of localized *Salmonella* infection, proper antibiotic therapy can decrease the mortality rate in these patients. We recommend further study of this role, since it may lead to a better understanding of the pathogenicity of the organism in these patients.

## Supplementary Data

Supplementary material 1
