# Supplementary material for: Pleural empyema due to Salmonella in a patient with bronchogenic carcinoma: the first case report from a cancer hospital in Egypt
Source: Access Microbiol. 2020 Jul 15;2(9):acmi000151. doi: 10.1099/acmi.0.000151 (PMC7656189; doi:10.1099/acmi.0.000151)
Supplement: Supplementary material 1 [file acmi-2-151-s001.pdf]

Microbiology Unit

Pleural Fluid Examination

Physical Examination:

- **Aspect:** Turbid – Greenish

Microscopic Examination:

- **Total Leucocytic Count:** 50.000 / cmm
- **ZN Stained Film:** Negative for AFB

Chemical Examination:

Glucose : 21 mg/dL

Total Protein: 4.5 g/dL

LDH: 23212 U/L

Culture:

Organism Identified ***Salmonella* group***

Antimicrobial	*Interpretation*	*MIC*(µg/ml)*
Piperacillin	S	≤ 4.0
Piperacillin/Tazobactam	S	≤ 4.0
Ceftazidime	S	≤ 1.0
Cefepime	S	≤ 1.0
Aztreonam	S	≤ 1.0
Imipenem	S	≤ 0.25
Meropenem	S	≤ 0.25
Ciprofloxacin	S	≤ 0.25
Trimethoprim/Sulfamethoxazole	S	≤ 20.0
Cefazolin	R	Inactive *in vivo*
Cefalexin	R	Inactive *in vivo*
Cefadroxil	R	Inactive *in vivo*
Cefuroxime	R	Inactive *in vivo*
Cefoxitin	R	Inactive *in vivo*
Cefotetan	R	Inactive *in vivo*
Cefprozil	R	Inactive *in vivo*

Cefaclor	R	Inactive *in vivo*
Amikacin	R	≥ 64.0
Gentamicin	R	≥ 16.0
Tobramycin	R	≥ 16.0
Benzylpenicillin	R	Intrinsic Resistant
Teicoplanin	R	Intrinsic Resistant
Vancomycin	R	Intrinsic Resistant
Azithromycin	R	Intrinsic Resistant
Clarythromycin	R	Intrinsic Resistant
Erythromycin	R	Intrinsic Resistant
Clindamycin	R	Intrinsic Resistant
Linezolid	R	Intrinsic Resistant
Daptomycin	R	Intrinsic Resistant
Rifampicin	R	Intrinsic Resistant
Fusidic Acid	R	Intrinsic Resistant

- **S: S**ensitive**, R: R**esistant**.**
- **MIC: M**inimum **I**nhibitory **C**oncentration**.**
